# Targeting Smoothened as a New Frontier in the Functional Recovery of Central Nervous System Demyelinating Pathologies

**DOI:** 10.3390/ijms19113677

**Published:** 2018-11-20

**Authors:** Alice Del Giovane, Antonella Ragnini-Wilson

**Affiliations:** Department of Biology University of Rome Tor Vergata, Viale Della Ricerca Scientifica, 00133 Rome, Italy; alice.delgiovane.adg@gmail.com

**Keywords:** remyelination, oligodendrocytes, drug screening, smoothened agonists

## Abstract

Myelin sheaths on vertebrate axons provide protection, vital support and increase the speed of neuronal signals. Myelin degeneration can be caused by viral, autoimmune or genetic diseases. Remyelination is a natural process that restores the myelin sheath and, consequently, neuronal function after a demyelination event, preventing neurodegeneration and thereby neuron functional loss. Pharmacological approaches to remyelination represent a promising new frontier in the therapy of human demyelination pathologies and might provide novel tools to improve adaptive myelination in aged individuals. Recent phenotypical screens have identified agonists of the atypical G protein-coupled receptor Smoothened and inhibitors of the glioma-associated oncogene 1 as being amongst the most potent stimulators of oligodendrocyte precursor cell (OPC) differentiation in vitro and remyelination in the central nervous system (CNS) of mice. Here, we discuss the current state-of-the-art of studies on the role of Sonic Hedgehog reactivation during remyelination, referring readers to other reviews for the role of Hedgehog signaling in cancer and stem cell maintenance.

## 1. Introduction

The Hedgehog (Hh) signaling pathway is an evolutionary conserved signal transduction pathway that plays a crucial role during embryonic development and tissue regeneration in vertebrates. Hh ligands, namely Sonic Hedgehog (Shh), Desert Hedgehog (Dhh) and Indian Hedgehog (Ihh), have similar binding affinities for membrane receptors called Patched (PTCH). Shh acts to establish cell fate in the developing limb, somites and the neuronal tube, Ihh is involved in chondrocyte development and Dhh in germ cell development. In the canonical Hh signaling pathway, the interaction of an Hh ligand with PTCH receptors activates the orphan G protein-coupled seven-pass transmembrane receptor Smoothened (Smo) in a chain of events that culminates in a change of balance between activator and repressor forms of the glioma-associated oncogenes (Gli1-3). In the absence of the Hh ligand, the Gli transcription factors are bound to Suppressor of Fused (SUFU). Upon Smo activation, the Gli proteins migrate to the nucleus where they regulate Hh-dependent gene transcription [1,2,3]. The Shh ligand is the most widely expressed across different tissues and its reactivation during CNS regenerative processes has been implicated in remyelination and adult white matter remodelling [2,4,5,6]. On the other hand, Hh signaling upregulation is also known to lead to cancer development and resistance [2,7].

Neurons depend on oligodendrocytes (OLs) for the release and re-uptake of neurotransmitters, neurotropic factors and metabolites but also for axon guidance and protection from oxidative stresses, just to name some of the many functions of glia/neuron association [8]. Thus, it is not surprising that axon demyelination results in neuronal degeneration, unless myelin is repaired [9]. The process of remyelination is active in healthy individuals until late stages of life but can fail in patients with multiple sclerosis (MS), with a progression that depends on the disease severity [10]. Advances in our understanding of myelin plasticity in the adult brain have been paralleled by the development of oligodendrocyte cell-based assays allowing for large screens of small molecules with regard to their promyelinating properties [11,12,13,14,15,16,17,18]. These studies identified a number of biologically active drugs with remyelination properties, including glucocorticoids acting as Smo agonists [14,15], imidazole antifungal drugs [14,17], Benztropine, an anticholinergic drug used in the treatment of Parkinson’s disease [12], epidermal growth factor receptor (EGFR) inhibitors [11,15], and sterol regulatory element binding (SREB) factors [18]. Of these, the most active drugs promoting remyelination in vivo were found to be the Smo agonist Clobetasol and the imidazole antifungal agent Miconazole [14,15,16]. This latter has been recently shown to act on OPC differentiation by downregulating the enzyme CYP51 of the cholesterol biosynthetic pathway [17]. These findings have been paralleled by evidence showing that Smo activity is regulated by cholesterol intermediates [19,20]. Moreover, upregulation of components of the Hh pathway is at the basis of several cancers [21,22,23] and antagonists of Smo/Gli signaling are potent anticancer agents in tumours refractory to current therapies [2,7]. These data highlight the need to clarify how the Shh/Smo/Gli signaling cascade regulates CNS regenerative properties in the adult brain, in order to develop therapies that retain the regenerative properties of Smo agonists but also reduce their tumorigenic potential.

Here, we review the main aspects of Shh signaling reactivation during remyelination and how Smo agonists promote OPC differentiation, taking into consideration recent structural and mechanistic studies on the mechanism of Smo activation.

## 2. Shh Signaling during CNS Remyelination

The process of CNS myelination begins during embryogenesis and is completed during adolescence. Secreted Shh is required during embryogenesis for the early appearance of OPCs in the spinal cord and forebrain and favours the maturation of OL neurospheres derived from embryonic rat brain [24]. Shh mouse mutants are defective in OPC differentiation to the mature phenotype and null mutations cause a severe holoprosencephaly (HPE) and defective induction of the floorplate of the entire neural tube [25,26].

In humans, myelin remains in place for life unless environmental or metabolic stress(es) or genetic dysfunction(s) damage its integrity [27,28,29]. After demyelination, remyelination restores the damaged myelin sheath surrounding the axon and prevents neurodegeneration [9,29,30]. Recent advances in in vivo neuroimaging techniques have provided the possibility to study white matter plasticity in the adult brain [30,31,32], showing that myelin regeneration is dynamically regulated after a demyelinating event [9] and during adaptive learning [28,31,32,33].

Shh morphogenetic signaling is not active in most CNS adult tissues but it is reactivated during remyelination [6,34]. Studies using the lysolecithin lysophosphatidyl-choline (LPC)-induced focal demyelination mouse model, and those using the cuprizone intoxication model showed that chondroitin sulphate proteoglycan-positive (NG2^+^) cells are recruited to areas of demyelination from the Sub Ventricular Zone (SVZ) following Shh signaling reactivation, and from there, migrate into the corpus callosum [35,36,37]. Shh reactivation regulates the neural stem cell (NSC) niches and its proliferation in the postnatal telencephalon [38], the adult hippocampus [39], and in the SVZ [6,34,40].

**Figure 1:**
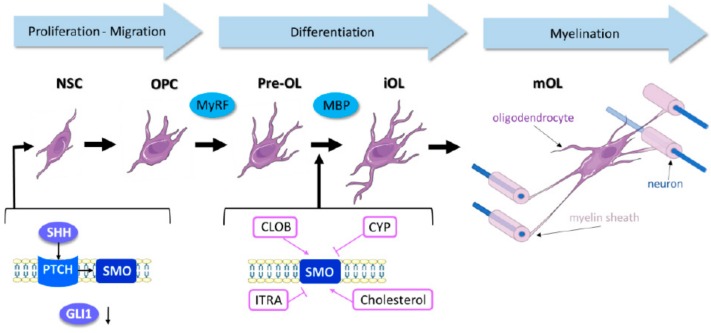
The role of Shh (Sonic Hedgehog) in neural stem cell (NSC) commitment to oligodendrocyte differentiation during remyelination. The process of oligodendrocyte maturation from NSCs to myelinating oligodendrocytes (mOL) requires Shh signaling reactivation. Smo activity seems to be crucial during the differentiation from premyelinating OLs (pre-OL) to immature oligodendrocytes (iOL). Smo agonists, such as Clobetasol (CLOB) or cholesterol, stimulate this passage while Smo inhibitors, such as cyclopamine (CYP) or itraconazole (ITRA), impair OL maturation via a poorly understood process.

Single-cell RNA sequencing of OPC subtypes obtained from OPCs derived from 10 different regions of mouse juvenile and adult CNS has shown that diverse subtypes of mature OLs are present in different CNS regions, although some populations are present in all regions. This high-resolution view of the transcriptional landscape of OPC maturation in the murine CNS showed that there are at least six distinct OL transcriptional phases, despite the fact that OPC maturation to OLs is a transcriptional continuum that can occur at different times of life in different brain regions [41] (Figure 1).

Shh upregulation is observed in NSCs of the OL lineage involved in lesion repair but not in normal adult white matter [6]. The Shh ligand during remyelination can be secreted by neurons and astrocytes [39,42,43,44,45]. Upregulation of Shh in astrocytes has been reported in an Experimental Autoimmune Encephalomyelitis (EAE) inflammatory model for MS and at lesions in MS patients compared to normal brain [44]. Further, supporting the role of Shh in NSC proliferation, thyroid hormone therapy is a potent inducer of oligodendrogenesis in remyelination animal models [46]. Brain delivery of Shh increases the number of OPCs and premyelinating OLs (pre-OLs)-expressing NG2 [47]. The emergence of OPCs from NSCs requires the expression of the oligodendrocyte lineage transcription factors 1 and 2 (Olig1 and Olig2), which is dependent on Shh. Olig2 function in adult OPCs is to accelerate the remyelination rate at demyelinated lesions by promoting OPC differentiation, as shown by the observation that Olig2 overexpression in LPC-induced demyelination is sufficient for enhancing OPC migration and differentiation, leading to precocious myelination [48]. Adenovirus-mediated transfer of Shh reduces inflammation and reactive astrogliosis while blocking Shh activity reduces the number of NG2^+^/Olig2^+^ cells-expressing Ki67, a marker of proliferating cells [47]. Deletion of the Shh ligand receptor PTCH in mouse astrocytes committed to OLs leads to an increase in the stemness of the NSCs without promoting tumour formation [35].

During remyelination, Shh signaling works in concert with epidermal growth factors (EGFs) and fibroblast growth factors (FGFs) that are among the major soluble factors regulating NSC re-entry into the cell cycle and the migration of neuroblasts [35,49]. Smo crosstalk with EGFR-mediated signaling has been highlighted in several tumours [22,50], but how EGFR and Shh signaling crosstalk to promote NSC differentiation towards the OPC lineage remains unclear. The proliferative effects of EGFR upregulation in Shh-reactivated NSCs have been suggested to be blocked by increasing NSC symmetrical cell division over the asymmetrical cell division via a mechanism that requires Shh expression [6,51].

Remyelination studies performed with LPC-induced focal demyelination animal models have indicated that the expression and transcriptional activation of several downstream components of Shh signaling is required for NSC differentiation to OPCs. PTCH, Smo, Gli1 and Gli2 are expressed in the area of the brain lesion at early stages of remyelination while Gli3 is not. Conditional inactivation of PTCH (Ptc) in mice enabled a study of the function of Shh signaling in the absence of a ligand in the SVZ. Interestingly, Ptc mutants showed an increase in EGFR expression, again supporting the view that Shh and EGFR signaling are co-regulated during remyelination [6,35].

Gli1 is considered as the canonical downstream effector of Shh/Smo signaling [2]. Gli1 is expressed only after sustaining Smo activation and conditional ablation of Smo in Gli1-expressing NSCs (Gli^CE/+^, Smo^FxFx^; RCE mice) does not increase the amount of NSCs or alter their cell fate, indicating that Gli1 downregulation upon Smo activation represents a specific signal that constrains NSC proliferation at remyelinating lesions. Unexpectedly, Smo activation in the absence of Gli1 function has even greater effects on NSC proliferation and increases NSC mobilization [52]. Previous studies have shown that Gli1 mRNA is upregulated in active lesions in EAE, but is significantly decreased in chronic active and inactive lesions in the MS brain compared to normal brain. Inflammatory cytokines such as IFN-γ can increase Shh in astroglia and NSCs, although they inhibit Gli1 in NSCs in the spinal cord after EAE onset. This observation led the authors to suggest that NSCs or OPCs proliferate in MS and EAE, but might be defective in maturation into OLs at lesions [44].

The Smo agonist SAG activates Hh signaling through both canonical and non-canonical pathways [21,53]. Microinjection of SAG into the corpus callosum of healthy or cuprizone-induced mice after chronic demyelination has been used to test if NSC mobilization could be achieved using ectopical in situ treatment with Smo agonists. An increase in Gli1 fate-labelled cells was detected in the adjacent V-SVZ but not in the corpus callosum of healthy adult mice [54]. By contrast, the corpus callosum exhibited recruitment and/or local activation of Gli1 after chronic demyelination consequent to cuprizone treatment [55].

In summary, accumulating data [6,35,44,50,52,54,55,56] show that NSCs fated to generate oligodendrocytes are preferentially located at the dorsolateral SVZ. NSC reactivation and proliferation rely not only on Shh upregulation and Smo signaling but also on concomitant Gli1 downregulation to allow for NSC differentiation toward the OL lineage (Figure 1).

## 3. Shh/Smo Signaling During OPC Differentiation

Much less is understood about the role of Shh/Smo signaling in the passage of OPCs from pre-OLs (Figure 1) to myelinating OLs [57]. At LPC-toxin-induced demyelination lesions, expression of the proteolipid protein (PLP), myelin basic protein (MBP) and 2′,3′-cyclic nucleotide 3′-phosphodiesterase (CNPase) begins within five days of toxin cessation, whereas OLs expressing myelin oligodendrocyte glycoprotein (MOG) start to appear eight weeks after the initiation of remyelination [58]. MBP-expressing OLs start to enlarge their membranes when MBP levels reach their maximal expression and via a signal that requires F-actin cytoskeleton depolymerisation. Membrane enlargement is initiated by cofilin and gelsolin release from their association with phosphatidylinositol 4,5-bisphosphate (PIP2) and binding of MBP to PIP2. These F-actin cystoskeletal changes are accompanied by cholesterol-mediated PLP trafficking to membranes [59,60]. Recent studies have shown that axon engagement by myelinating OLs does not require neuronal feedback at early stages, since OL lineage-specific cues define the length and dimension of myelin wrapping around axons [61]. The early stages of axon engagement depend mainly on axon fiber diameter since the presence of inert polystyrene microfibers of 2–4 µm are sufficient to initiate myelination in the presence of mature OLs [62].

The potential requirement of Smo activation for the transition of pre-OLs into mature OLs expressing myelin genes is suggested by the finding that two glucocorticoids that act as Smo agonists [21], Clobetasol and Halcinonide, promote MBP expression in the immortalized oligodendrocyte mouse cell line Oli-neu that expresses MyRF (Oli-neuM) [15]. Clobetasol treatment promotes remyelination in EAE and neuromyelitis optica mouse models [14,16]. MyRF is a membrane-associated transcription factor that enhances the differentiation of OPCs when overexpressed [63,64]. OPC-specific *MyRF* gene deletion did not alter recruitment or initial differentiation of OPCs in LPC-induced demyelination of the corpus callosum in mice but decreased the density of new glutathione *S*-transferase π-positive oligodendrocytes and impaired remyelination in the spinal cord and corpus callosum, showing incapacity to express myelin proteins [64]. Extracellular signal-regulated kinases 1/2 (ERK1/2) regulates *MyRF* gene expression in OLs during development, and *MyRF* gene expression activates a cascade of events, leading to axon engagement in the healthy CNS. Interestingly, it has been observed that conditional ablation of *MyRF* in the brain leads to an inability of mice to adapt to environmental changes required for adaptive learning, a process that requires myelination of novel axons [33]. Supporting these data, Smo inhibition by cyclopamine impairs OPC differentiation to myelinating OLs and drug removal restores *MBP* and *MAG* gene expression in primary OPCs [57]. Clobetasol-dependent *MBP* gene expression and the morphological changes accompanying Oli-neuM differentiation upon Clobetasol treatment require Smo activation and *RxRγ* gene transcription as cyclopamine or itraconazole treatment reduces *MBP* expression in Clobetasol-treated Oli-neuM and RxRγ inhibition in Clobetasol-treated Oli-neuM results in MBP downregulateion [15]. RxRγ can form homodimers or heterodimers with other nuclear receptors (RAR, RxR, VxD or PPARs) depending on the ligand stimulation [65]. *RxRγ* gene expression is upregulated at remyelination lesions in MS patients and its downregulation in cultured OPCs results in depletion of differentiated OLs in purified OPC cultures, with OLs stalled at the premyelination stage. However, neither the ligand that stimulates its transcription nor its receptor-binding partner has been identified under remyelination stimuli [66].

## 4. Smo and Gli-Associated Oncogene Regulation in Adult Somatic Cells

### 4.1. Smo and Gli Antagonists

The finding that Shh signaling is activated in cancer cells has powered the search for Smo or Gli antagonists [7,67,68,69,70,71,72]. Among them are the natural alkaloid cyclopamine [21] and the antifungal agents itraconazole [21] and SANT1 [70], LY2940680 [71] and Vismodegib [72]. The sterol alkaloid cyclopamine has been largely used in studies addressing the function of Smo in NSC proliferation [6,35] and OPC differentiation [15,57]. Cyclopamine binds to the extracellular end of the Seven Transmembrane (7TM) domain and to the cysteine-rich domain (CRD) of Smo [73,74] and competes for the binding site of the synthetic Smo agonist SAG [75,76]. Cyclopamine impairs Smo activity upon Shh binding to PTCH, despite shown agonistic properties since it does not impair Smo translocation at the cilium, considered as a hallmark of Smo activation [19,74]. Cyclopamine injection into lateral ventricles aggravates ischemic brain damage and the Smo agonist purmorphamine acts as a neuroprotective agent in a model for ischemic injury [8]. Itraconazole is an antifungal agent that antagonises Smo with antiproliferative activity on several cancers, among which are glioma and medulloblastoma when administrated systemically, and suppresses the growth of basal cell carcinoma of skin [76]. Itraconazole fails to compete with BODIPY-cyclopamine, a fluorescent derivative of cyclopamine that binds Smo and inhibits Shh signaling, indicating that it acts at a different site compared with cyclopamine. Interestingly, unlike cyclopamine, itraconazole prevents Smo ciliary accumulation and acts as a non-competitive inhibitor of the synthetic Smo agonist SAG [77].

Staurosporinone, zerumbone, arcyriaflavin C, physalin B and physalin F can effectively inhibit both Gli1- and Gli2-mediated transcription. GANT61 has been tested for its therapeutic potential in the EAE model for relapsing remitting MS [52] by specifically targeting the Gli1 transcription factor. Other synthetic Gli inhibitor agents are GANT58, HPI1-4, ATO, GlaB, JQ1 and I-BET151 each with a different mode of action. GANT58 and GANT61 impair Gli binding to DNA and the others mainly affect Gli1/2 phophorylation [78]. These inhibitors are used to clarify the role of Gli proteins in canonical and non-canonical Shh/Smo signaling.

### 4.2. Canonical Pathways of Gli Regulation

The canonical Shh signal relies on Smo translocation to the tip of the primary cilium to change the balance of Gli transcriptional activators (GliA), primarily Gli2A, and Gli transcriptional repressors (GliR, [67,79,80]). Rat OPCs at early stages of migration and oligodendrocyte differentiation display markers of the primary cilium (e.g. γ-tubulin, glutamylated tubulin, acetylated tubulin, and ADP-ribosylation factor-like 13B). However, the precise role of the primary cilium in Smo signaling during OPC differentiation remains to be established [81]. Reporter gene assays and analysis of marker gene expression in transgenic and mutant animals have demonstrated that Gli1 functions as a strong transcriptional activator while Gli3 mainly acts as a transcriptional repressor. Gli2 can have positive as well as negative effects on gene transcription. The N-terminal repressor domain, not present in the Gli1 protein, mediates Gli2 and Gli3 binding with the cytoplasmic protein SUFU [3,82]. SUFU controls Gli(s) nuclear entry and thereby their transcriptional effects, and SUFU mutation in germline or somatic cells can lead to meningioma and chondrosarcoma [50,82]. Binding of Gli proteins to promoters initiates the downstream Shh signaling leading to the expression, among others, of the main target genes of the Hh signaling pathway, such as PTCH1, PTCH2, and Gli1 [3].

The *Gli1* gene is required for Hh signaling in zebra fish [83] but not in mice where its deletion is viable [84], although Gli1 mutants have defects in Shh signaling in combination with a Gli2 mutation [85,86]. Recently, the recessive Ellis–van Creveld syndrome (EvC; MIM: 225500) has been associated with a truncated *Gli1* gene [87]. Gli2 mutant phenotypes can be rescued by *Gli1* gene insertion into the Gli2 locus, while Gli3 cannot [84]. The mouse Gli2 transcriptional activity is distinct from that of Gli3, although Gli2 and Gli3 share 44% of overall amino acid identity, sequence similarity in the activator and repressor domain and conserved PKA sites [87]. Degradation of Gli2 is regulated by thephosphorylation of a cluster of four PKA sites within the Gli2 C-terminal region. Moreover, PKA primes further phosphorylation by GSK3 and CK1 [88]. Gli2 is mainly regarded as a transcriptional activator and loss of Gli2 results in defects in floorplate induction. The hyperphosphorylation of the Gli2 protein in turn conjugates multiple ubiquitin molecules onto the Gli2 protein and triggers its proteasome-mediated protein degradation [89].

Heterozygous mutations in Gli3 account for several dominant diseases of variable severity, including Greig cephalopolysyndactyly syndrome (GCPS; MIM: 175700) [90,91], Pallister-Hall syndrome (MIM: 146510) [92], preaxial polydactyly type IV (MIM: 174700) and postaxial polydactyly types A1 and B (MIM: 174200) [93,94]. Gli2 heterozygous mutations are associated with holoprosencephaly (HPE9; MIM: 610829) and Culler-Jones syndrome (MIM: 615849) [95,96].

### 4.3. Non-Canonical Pathways of Gli Regulation

The finding that Gli1 is downregulated in Shh-responsive NSCs suggested that a non-canonical pathway of Gli1 inactivation might be activated in Shh-responsive NSCs originating from the SVZ during remyelination [52]. So far, non-canonical Gli activation signaling has been investigated mainly in the context of malignant diseases [7,78]. With the exception of Notch signaling which interferes with the Shh ligand, the RAS/RAF/MEK/ERK-, PI3K/AKT/mTOR- and EGFR-mediated signaling pathways have been shown to crosstalk with the Hh pathway by interfering with Gli activity.

Gli proteins, mainly Gli1, have been reported to be activated by AKT [97,98], MAPK/ERK [99], and KRAS [100] in an Hh ligand–PTCH1–Smo axis-independent or a Smo-independent manner [101]. PI3K/AKT and MEK/ERK pathways cooperate with Hh at the level of Gli1 to promote proliferation and survival of esophageal cancer cells. In a second model of esophageal cancer, activated mTOR/S6K1 was shown to phosphorylate Gli1, that consequently releases SUFU, and activates Gli1 target gene transcription, enhancing oncogenic function [102]. In keratinocytes, ERK1/2 activated by EGF stabilizes Gli proteins, particularly Gli2. EGFR and *Gli* genes have also been shown to negatively regulate one another and EGFR signaling leads to decreased expression of Hh signaling components, whereas inhibition of Hh signaling leads to increased EGFR signaling [103]. The KRAS–MEK–ERK cascade has been shown to regulate positively *Gli1* gene transcription either by preventing Gli1 protein degradation or by acting on Gli1 phosphorylation. The activation of Hh signaling in pancreatic cancer cells has been reported to be consequent to the block of the proteasome-mediated Gli1 degradation caused by oncogenic Kras. In human keratinocytes, Gli1 and Gli2 are stabilized by preventing their degradation via the proteasome pathway by EGFR-activated ERK1/2 [104].

The relationship between mTOR/S6K1 signaling and myelination is indicated by the observation that deletion of Raptor, Rheb1 or mTORC1 causes hypomyelination and reduction of OLs and an accumulation of OPCs in the spinal cord [105,106,107,108]. Moreover, rapamycin, an mTOR inhibitor, impairs the progression of O4-positive OPCs to GalC-positive OPCs in vitro [109]. Intriguingly, mTOR is implicated in sphingolipid metabolism in yeast [110] and Shh biosynthesis requires cholesterol [111]. Crosstalk between PI3K/mTOR and Hh signaling pathways occurs frequently in gastrointestinal cancers and co-treatment with rapamycin and vismodegib, inhibitors of the respective pathway, have shown efficacy in biliary tract cancer inhibition and in suppressesing cancer stem cell proliferation [108]. Riobo and colleagues demonstrated that activation of PI3-kinase/Akt increases Shh-induced Gli1 transcriptional activity through antagonizing PKA-dependent Gli2 inactivation in several experimental systems [112].

AMP-activated Protein Kinase (AMPK) signaling has a protective role for OLs under pathological conditions and delays disease progression in EAE. Interestingly, Metformin, an AMPK activator, attenuates increased inflammation and demyelination in the CNS compartment of the EAE animal model [113]. Gli1 activity can be also regulated by AMPK in medulloblastoma. AMPK phosphorylates directly Gli1 at serines 102 and 408 and threonine 1074 in the NIH-3T3 cell line, which is known to respond to Hh signaling. AMPK-dependent Gli1 phosphorylation leads to suppression of Gli1 transcriptional activity. This regulation slows down or postpones developmental steps dependent on Hh signaling when energy stores are inadequate in cells or organs, with the effects of enhancing survival [113].

TGF and its receptors are widely expressed in the human body, and its signaling plays a major role in human diseases including multiple sclerosis. There is increasing evidence to show that TGF signal transduction interacts with the Hh pathway downstream of Smo not only in normal fibroblasts and keratinocytes but also in various cancer cell lines. The TGF-/smad3 cascade results in Gli2 induction via a mechanism independent of the Hh/Ptch/Smo axis and does not require de novo protein synthesis [80]. In pancreatic ductal adenocarcinoma cancer cells lacking Smo, TGF beta treatment leads to marked elevation of Gli1 and Gli3, even when Gli2 expression is undetectable [100].

Notch activity affects the trafficking of Smo and PTCH1 to primary cilia, suggesting its interference in the transmission of Smo signaling [92]. The Notch signaling pathway regulates neuronal precursor cell maintenance and neuronal and glial development and is considered crucial for the development and clinical progression of MS. Notch activity enhances Shh signaling and Shh signaling induces expression of Notch ligands, indicating a crosstalk between these two pathways leading to OPC differentition [114,115], and Notch receptors are expressed at demyelinating lesions of the EAE animal model [116]. 

## 5. Shh Signaling, Cholesterol Biosynthesis and Myelination: A Complex Liaison

Shh ligand formation depends on cholesterol for its biosynthesis, and cholesterol is necessary for the expression of genes that encode myelin proteins since mutations that affect cholesterol biosynthesis cause hypomyelination and reduce levels of myelin gene transcripts [117]. Similarly, statin treatment promoting pharmacological inhibition of cholesterol synthesis reduces the amount of myelin gene transcripts. Clearly, several molecular mechanisms connect cholesterol to Smo activation and myelination. Two recent phenotypical screens for drugs promoting myelination have highlighted a further unexpected role of cholesterol and cholesterol intermediates in promoting OPC differentiation until axon engagement. Interestingly, two compounds identified for their ability to promote oligodendrocyte differentiation and remyelination, Miconazole and Clotrimazole [14,15], have been shown to impinge on cholesterol metabolism to activate the signals leading to remyelination [17]. The third most active compound identified so far for its remyelination properties, Clobetasol, is a Smo agonist [14,15,21]. Consistent with the idea that they act through a common mechanism that involves cholesterol biosynthesis or cholesterol mediated regulation of differentiation, a comparative transcriptome analysis of mouse epiblast stem cell-derived OPCs (mEpiSC-OPCs) treated with Miconazole or Clobetasol identified the sterol regulatory element binding factors (SREBFs) family of transcription factors among the commonly expressed genes [18]. Ashikawa and colleagues show that SREB activation increases the expression of 3-hydroxy-3-methylglutaryl-CoA reductase (HMGCR; [118,119]), stearoyl-CoA desaturase (SCD) [120], cytochrome P450 family 51 subfamily A polypeptide 1 (CYP51A1) [121], acyl-CoA synthetase short-chain family member 2 (ACSS2) [122], and 7-dehydrocholesterol reductase (DHCR7) [123]. SREBs are regulators of sterol biosynthesis [124] and myelination [125]. Consistent with this finding, mice lacking the SREBP-controlled squalene synthase in Schwann cells are affected by severe hypomyelination [126]. Furthermore, several studies have shown that PI3K/Akt/mTOR signaling promotes cholesterol biosynthetic pathway gene expression through activation of the SREBP [107,109,127,128] and SREB regulation depends on *RxRs* and *LxR* gene expression. Thus, there exists a clear relationship among myelination, cholesterol biosynthesis, Clobetasol-mediated MBP expression and Shh signaling activation during OPC differentiation into myelinating OLs, but the molecular players that connect Smo activation to myelination during OPC maturation remain to be identified.

The Shh ligand is a soluble factor that originates from the cholesterol-mediated autocatalytic cleavage of the Shh precursor [111]. In the ER, the Shh precursor undergoes a cholesterol-dependent autoproteolytic cleavage, generating one N-terminal fragment (N-Shh), containing the Hedge domain, linked to cholesterol, with ligand properties and a C-Shh domain that is degraded. After cholesterol addition and palmytolation, N-Shh hydrophobicity and secretion increases [129] and the post-translationally modified N-Shh fragment becomes an active Shh ligand that can bind to PTCH (PTCH1 and PTCH2) receptors that, unless bound to the ligand, act as Smo inhibitors [2]. Based on their differential expression during epidermal development, *PTCH1* and *PTCH2* genes have likely different functions. Two PTCH receptors bind one Shh ligand [130].

How the Shh signal is transduced from PTCH to Smo remained unclear for a long time. Recent structural studies have clarified that PTCH suppresses the activity of Smo by impairing conformational changes induced by sterol binding [20,130]. It has been observed that PTCH1 has homology to a lysosomal cholesterol transporter, the Niemann-Pick C1 (NPC1) protein [131] that binds and transports cholesterol [132]. NPC1 loss of function in humans leads to the Niemann-Pick type C disease, a childhood-onset neurodegenerative disorder characterized by intracellular lipid accumulation, abnormally swollen axons, and neuron loss. In NPC patients, CNS hypomyelination is observed and mice lacking Npc1, in either neurons or oligodendrocytes, exhibit a defect in myelin formation in selected regions of the brain caused by arrest in oligodendrocyte maturation [133]. Lucchetti and colleagues [19] have proposed that PTCH1, due to its potential cholesterol-binding ability, may inhibit Smo by reducing cholesterol content or cholesterol accessibility at membrane compartments, leading to alterations in Smo conformation or trafficking [132,134,135]. It is also of note that an acute increase in plasma membrane cholesterol is sufficient to activate Hh signaling and pharmacological or genetic depletion reduces cellular responses to Hh ligands [19,136].

The crystal structure of cholesterol-bound Smo has been recently reported [19]. These studies showed that Smo activation is mediated through two different regions of the molecule. Cholesterol present in the plasma membrane activates Smo by binding to the extracellular cysteine-rich domain (CRD) and competes with another natural Smo agonist such as 20(*S*)hydroxycholesterol, and CRD mutations that abolish binding to cholesterol impair Smo activation [74,137]. This interaction opens a hydrophobic tunnel that leaves a path for cholesterol movement from the inner membrane leaflet to the CRD, as shown by the comparison of the cholesterol-bound structure with a structure of inactive Smo bound to the Smo antagonist Vismodegib (which lacks cholesterol in the CRD groove [72]). The comparison of Smo–cholesterol binding state with the Smo–Vismodegib conformation finally revealed the conformational change that drives Smo activation [20].

The fact that Shh processing and ligand formation requires cholesterol as well as the fact that Smo activation depends on cholesterol binding suggests that cholesterol abundance and accessibility at OPC membranes might have a regulatory role in NSC proliferation and OPC differentiation from pre-OLs to myelinating OLs. This would represent an additional level or regulation of OPC maturation into myelinating OLs during remyelination processes.

Indeed, several genetic disorders of the cholesterol biosynthetic pathway are associated with demyelination with or without craniofacial malformations and accumulation of sterol intermediate precursors [117,138]. OLs produce the bulk of cholesterol incorporated into myelin and, in the case of a deficiency in cholesterol biosynthesis, an efficient horizontal transfer of cholesterol between different brain cell types has been observed. Mice with cell type-specific inactivation of the *SQS* gene (Fdft1), an essential enzyme in cholesterol biosynthesis, show severe perturbation of myelin synthesis by OLs and a reduced rate of myelination in white matter, although purified myelin from SQS mutant mice has an almost normal composition of proteins and lipids, including the characteristic high cholesterol level [117]. Thus, the availability of cholesterol appears to be an essential and rate-limiting factor for myelin growth [117], Smo activation [19,20] and OPC differentiation into mature OLs [15,17].

## 6. Concluding Remarks

How Shh reactivation leads to NSC differentiation toward the OPC lineage during remyelination processes is beginning to be clarified [6,35,36,37,38,39,40,41,42,43,48,52] and a number of drugs stimulating Smo activity and remyelination have been selected in large phenotypical screens, aiming at recovering the effects of pathological demyelination in adult brain [11,12,13,14,15,18]. In addition, several Gli1 inhibitors have been tested for their anticancer properties and their use in vitro and in vivo have helped to elucidate the basic question of how Shh signaling is regulated during remyelination as well as the structural features of how Shh signaling is transmitted to downstream effectors [23,74,75,76,77]. All these drugs represent powerful tools for anticancer and regenerative medicine research, but their use must take into consideration the delicate balance between GliA and GliR regulation by canonical and non-canonical activation signals. A lot still has to be done to clarify this part of Shh signaling during remyelination processes. The development of oligodendroglia supports that make use of nanofibers should help to clarify the mechanistic aspects of Smo activation/inactivation at the last step of myelin formation and during axon engagement [17,30,62]. The next frontier will be to create a 3D representation of neuronal/glia interaction in organoids to have a 3D vision of how Shh reactivation leads to remyelination.

## References

[1] Varjosalo M., Taipale J. (2008). Hedgehog: Functions and Mechanisms. Genes Dev..

[2] Rimkus T.K., Carpenter R.L., Qasem S., Chan M., Lo H.-W. (2016). Targeting the Sonic Hedgehog Signaling Pathway: Review of Smoothened and GLI Inhibitors. Cancers.

[3] Skoda A.M., Simovic D., Karin V., Kardum V., Vranic S., Serman L. (2018). The Role of the Hedgehog Signaling Pathway in Cancer: A Comprehensive Review. Bosn. J. Basic Med. Sci..

[4] Carpenter D., Stone D.M., Brush J., Ryan A., Armanini M., Frantz G., Rosenthal A., De Sauvage F.J. (1998). Characterization of Two Patched Receptors for the Vertebrate Hedgehog Protein Family. Proc. Natl. Acad. Sci. USA.

[5] Buglino J.A., Resh M.D. (2008). Hhat Is a Palmitoylacyltransferase with Specificity for N-Palmitoylation of Sonic Hedgehog. J. Biol. Chem..

[6] Ferent J., Zimmer C., Durbec P., Ruat M., Traiffort E. (2013). Sonic Hedgehog Signaling Is a Positive Oligodendrocyte Regulator during Demyelination. J. Neurosci..

[7] Infante P., Mori M., Alfonsi R., Ghirga F., Aiello F., Toscano S., Ingallina C., Siler M., Cucchi D., Po A. (2015). Gli1/DNA Interaction Is a Druggable Target for Hedgehog-Dependent Tumors. EMBO J..

[8] Chew L.-J., DeBoy C.A. (2016). Pharmacological Approaches to Intervention in Hypomyelinating and Demyelinating White Matter Pathology. Neuropharmacology.

[9] Kremer D., Göttle P., Hartung H.-P., Küry P. (2016). Pushing Forward: Remyelination as the New Frontier in CNS Diseases. Trends Neurosci..

[10] Villoslada P. (2016). Neuroprotective Therapies for Multiple Sclerosis and Other Demyelinating Diseases. Mult. Scler. Demyelinating Disord..

[11] Joubert L., Foucault I., Sagot Y., Bernasconi L., Duval F., Alliod C., Frossard M.-J., Pescini Gobert R., Curchod M.-L., Salvat C. (2010). Chemical Inducers and Transcriptional Markers of Oligodendrocyte Differentiation. J. Neurosci. Res..

[12] Deshmukh V.A., Tardif V., Lyssiotis C.A., Green C.C., Kerman B., Kim H.J., Padmanabhan K., Swoboda J.G., Ahmad I., Kondo T. (2013). A Regenerative Approach to the Treatment of Multiple Sclerosis. Nature.

[13] Mei F., Fancy S.P.J., Shen Y.A., Niu J., Zhao C., Presley B., Miao E., Lee S., Mayoral S.R., Redmond S.A. (2014). Micropillar arrays as a high-throughput screening platform for therapeutics in multiple sclerosis. Nat. Med..

[14] Najm F.J., Madhavan M., Zaremba A., Shick E., Karl R.T., Factor D.C., Miller T.E., Nevin Z.S., Kantor C., Sargent A. (2015). Drug-Based Modulation of Endogenous Stem Cells Promotes Functional Remyelination In Vivo. Nature.

[15] Porcu G., Serone E., De Nardis V., Di Giandomenico D., Lucisano G., Scardapane M., Poma A., Ragnini-Wilson A. (2015). Clobetasol and Halcinonide Act as Smoothened Agonists to Promote Myelin Gene Expression and RxRγ Receptor Activation. PLoS ONE.

[16] Yao X., Su T., Verkman A.S. (2016). Clobetasol Promotes Remyelination in a Mouse Model of Neuromyelitis Optica. Acta Neuropathol. Commun..

[17] Hubler Z., Allimuthu D., Bederman I., Elitt M.S., Madhavan M., Allan K.C., Shick H.E., Garrison E., Karl M., Factor D.C. (2018). Accumulation of 8,9-Unsaturated Sterols Drives Oligodendrocyte Formation and Remyelination. Nature.

[18] Ashikawa Y., Nishimura Y., Okabe S., Sasagawa S., Murakami S., Yuge M., Kawaguchi K., Kawase R., Tanaka T. (2016). Activation of Sterol Regulatory Element Binding Factors by Fenofibrate and Gemfibrozil Stimulates Myelination in Zebrafish. Front. Pharmacol..

[19] Luchetti G., Sircar R., Kong J.H., Nachtergaele S., Sagner A., Byrne E.F., Covey D.F., Siebold C., Rohatgi R. (2016). Cholesterol Activates the G-Protein Coupled Receptor Smoothened to Promote Hedgehog Signaling. eLife.

[20] Huang P., Zheng S., Wierbowski B.M., Kim Y., Nedelcu D., Aravena L., Liu J., Kruse A.C. (2018). Structural Basis of Smoothened Activation in Hedgehog Signaling. Cell.

[21] Wang J., Lu J., Bond M.C., Chen M., Ren X.-R., Lyerly H.K., Barak L.S. (2010). Identification of Select Glucocorticoids as Smoothened Agonists: Potential Utility for Regenerative Medicine. Proc. Natl. Acad. Sci. USA.

[22] Mangelberger D., Kern D., Loipetzberger A., Eberl M., Aberger F. (2012). Cooperative Hedgehog-EGFR Signaling. Front. Biosci..

[23] Dirix L. (2014). Discovery and Exploitation of Novel Targets by Approved Drugs. J. Clin. Oncol..

[24] Gibney S.M., McDermott K.W. (2009). Sonic hedgehog promotes the generation of myelin proteins by transplanted oligosphere-derived cells. J. Neurosci. Res..

[25] Roessler E., Belloni E., Gaudenz K., Jay P., Berta P., Scherer S.W., Tsui L.C., Muenke M. (1996). Mutations in the human Sonic Hedgehog gene cause holoprosencephaly. Nat. Genet..

[26] Bertolacini C.D., Richieri-Costa A., Ribeiro-Bicudo L.A. (2010). Sonic hedgehog (SHH) mutation in patients within the spectrum of holoprosencephaly. Brain Dev..

[27] Deoni S.C.L., Mercure E., Blasi A., Gasston D., Thomson A., Johnson M., Williams S.C.R., Murphy D.G.M. (2011). Mapping Infant Brain Myelination with Magnetic Resonance Imaging. J. Neurosci..

[28] Wang S., Young K.M. (2014). White Matter Plasticity in Adulthood. Neuroscience.

[29] Almeida R.G., Lyons D.A. (2017). On Myelinated Axon Plasticity and Neuronal Circuit Formation and Function. J. Neurosci..

[30] Auer F., Vagionitis S., Czopka T. (2018). Evidence for Myelin Sheath Remodeling in the CNS Revealed by In Vivo Imaging. Curr. Biol..

[31] Tomassy G.S., Dershowitz L.B., Arlotta P. (2016). Diversity Matters: A Revised Guide to Myelination. Trends Cell Biol..

[32] Kaller M.S., Lazari A., Blanco-Duque C., Sampaio-Baptista C., Johansen-Berg H. (2017). Myelin Plasticity and Behaviour-Connecting the Dots. Curr. Opin. Neurobiol..

[33] McKenzie I.A., Ohayon D., Li H., De Faria J.P., Emery B., Tohyama K., Richardson W.D. (2014). Motor Skill Learning Requires Active Central Myelination. Science.

[34] Ihrie R.A., Shah J.K., Harwell C.C., Levine J.H., Guinto C.D., Lezameta M., Kriegstein A.R., Alvarez-Buylla A. (2011). Persistent Sonic Hedgehog Signaling in Adult Brain Determines Neural Stem Cell Positional Identity. Neuron.

[35] Ferent J., Cochard L., Faure H., Taddei M., Hahn H., Ruat M., Traiffort E. (2014). Genetic Activation of Hedgehog Signaling Unbalances the Rate of Neural Stem Cell Renewal by Increasing Symmetric Divisions. Stem Cell Rep..

[36] Zuccaro E., Arlotta P. (2013). The Quest for Myelin in the Adult Brain. Nat. Cell Biol..

[37] Daynac M., Pineda J.R., Chicheportiche A., Gauthier L.R., Morizur L., Boussin F.D., Mouthon M.A. (2014). TGFβ lengthens the G1 phase of stem cells in aged mouse brain. Stem Cells.

[38] Tekki-Kessaris N., Woodruff R., Hall A.C., Gaffield W., Kimura S., Stiles C.D., Rowitch D.H., Richardson W.D. (2001). Hedgehog-dependent oligodendrocyte lineage specification in the telencephalon. Development.

[39] Lai K., Kaspar B.K., Gage F.H., Schaffer D.V. (2003). Sonic Hedgehog Regulates Adult Neural Progenitor Proliferation in Vitro and In Vivo. Nat. Neurosci..

[40] Ahn S., Joyner A.L. (2005). In Vivo Analysis of Quiescent Adult Neural Stem Cells Responding to Sonic Hedgehog. Nature.

[41] Marques S., Zeisel A., Codeluppi S., Van Bruggen D., Mendanha Falcão A., Xiao L., Li H., Häring M., Hochgerner H., Romanov R.A. (2016). Oligodendrocyte Heterogeneity in the Mouse Juvenile and Adult Central Nervous System. Science.

[42] Charytoniuk D., Traiffort E., Hantraye P., Hermel J.M., Galdes A., Ruat M. (2002). Intrastriatal Sonic Hedgehog Injection Increases Patched Transcript Levels in the Adult Rat Subventricular Zone. Eur. J. Neurosci..

[43] Machold R., Hayashi S., Rutlin M., Muzumdar M.D., Nery S., Corbin J.G., Gritli-Linde A., Dellovade T., Porter J.A., Rubin L.L. (2003). Sonic Hedgehog Is Required for Progenitor Cell Maintenance in Telencephalic Stem Cell Niches. Neuron.

[44] Wang Q., Huang S., Yang L., Zhao L., Yin Y., Liu Z., Chen Z., Zhang H. (2008). Down-Regulation of Sonic Hedgehog Signaling Pathway Activity Is Involved in 5-Fluorouracil-Induced Apoptosis and Motility Inhibition in Hep3B Cells. Acta Biochim. Biophys. Sin..

[45] Sirko S., Behrendt G., Johansson P.A., Tripathi P., Costa M., Bek S., Heinrich C., Tiedt S., Colak D. (2013). Reactive glia in the injured brain acquire stem cell properties in response to sonic hedgehog. Cell Stem Cell.

[46] Harsan L.-A., Steibel J., Zaremba A., Agin A., Sapin R., Poulet P., Guignard B., Parizel N., Grucker D., Boehm N. (2008). Recovery from Chronic Demyelination by Thyroid Hormone Therapy: Myelinogenesis Induction and Assessment by Diffusion Tensor Magnetic Resonance Imaging. J. Neurosci..

[47] Loulier K., Ruat M., Traiffort E. (2006). Increase of Proliferating Oligodendroglial Progenitors in the Adult Mouse Brain upon Sonic Hedgehog Delivery in the Lateral Ventricle. J. Neurochem..

[48] Wegener A., Deboux C., Bachelin C., Frah M., Kerninon C., Seilhean D., Weider M., Wegner M., Nait-Oumesmar B. (2015). Gain of Olig2 Function in Oligodendrocyte Progenitors Promotes Remyelination. Brain.

[49] Angot E., Loulier K., Nguyen-Ba-Charvet K.T., Gadeau A.-P., Ruat M., Traiffort E. (2008). Chemoattractive Activity of Sonic Hedgehog in the Adult Subventricular Zone Modulates the Number of Neural Precursors Reaching the Olfactory Bulb. Stem Cells.

[50] Breachbiel J., Miller-Moslin K., Adjei A.A. (2014). Crosstalk between hedgehog and other signaling pathways as a basis for combination therapies in cancer. Cancer Treat. Rev..

[51] Sun Y., Goderie S.K., Temple S. (2005). Asymmetric distribution of EGFR receptor during mitosis generates diverse CNS progenitor cells. Neuron.

[52] Samanta J., Grund E.M., Silva H.M., Lafaille J.J., Fishell G., Salzer J.L. (2015). Inhibition of Gli1 mobilizes endogenous neural stem cells for remyelination. Nature.

[53] Briscoe J., Thérond P.P. (2013). The Mechanisms of Hedgehog Signaling and Its Roles in Development and Disease. Nat. Rev. Mol. Cell Biol..

[54] Mierzwa A.J., Sullivan G.M., Beer L.A., Ahn S., Armstrong R.C. (2014). Comparison of cortical and white matter traumatic brain injury models reveals differential effects in the subventricular zone and divergent Sonic hedgehog signaling pathways in neuroblasts and oligodendrocyte progenitors. ASN Neuro.

[55] Sanchez M.A., Armstrong R.C. (2018). Postnatal Sonic Hedgehog (Shh) Responsive Cells Give Rise to Oligodendrocyte Lineage Cells during Myelination and in Adulthood Contribute to Remyelination. Exp. Neurol..

[56] Gorojankina T., Hoch L., Faure H., Roudaut H., Traiffort E., Schoenfelder A., Girard N., Mann A., Manetti F., Solinas A. (2013). Discovery, Molecular and Pharmacological Characterization of GSA-10, a Novel Small-Molecule Positive Modulator of Smoothened. Mol. Pharmacol..

[57] Wang L.-C., Almazan G. (2016). Role of Sonic Hedgehog Signaling in Oligodendrocyte Differentiation. Neurochem. Res..

[58] Patel J.R., Klein R.S. (2011). Mediators of Oligodendrocyte Differentiation during Remyelination. FEBS Lett..

[59] Nawaz S., Sánchez P., Schmitt S., Snaidero N., Mitkovski M., Velte C., Brückner B.R., Alexopoulos I., Czopka T., Jung S.Y. (2015). Actin Filament Turnover Drives Leading Edge Growth during Myelin Sheath Formation in the Central Nervous System. Dev. Cell.

[60] Zuchero J.B., Fu M.-M., Sloan S.A., Ibrahim A., Olson A., Zaremba A., Dugas J.C., Wienbar S., Caprariello A.V., Kantor C. (2015). CNS Myelin Wrapping Is Driven by Actin Disassembly. Dev. Cell.

[61] Bechler M.E., Byrne L. (2015). CNS Myelin Sheath Lengths Are an Intrinsic Property of Oligodendrocytes. Curr. Biol..

[62] Lee S., Leach M.K., Redmond S.A., Chong S.Y.C., Mellon S.H., Tuck S.J., Feng Z.-Q., Corey J.M., Chan J.R. (2012). A Culture System to Study Oligodendrocyte Myelination Processes Using Engineered Nanofibers. Nat. Methods.

[63] Emery B., Agalliu D., Cahoy J.D., Watkins T.A., Dugas J.C., Mulinyawe S.B., Ibrahim A., Ligon K.L., Rowitch D.H., Barres B.A. (2009). Myelin Gene Regulatory Factor Is a Critical Transcriptional Regulator Required for CNS Myelination. Cell.

[64] Duncan G.J., Plemel J.R., Assinck P., Manesh S.B., Muir F.G.W., Hirata R., Berson M., Liu J., Wegner M., Emery B. (2017). Myelin regulatory factor drives remyelination in multiple sclerosis. Acta Neuropathol..

[65] Giner X.C., Cotnoir-White D., Mader S., Lévesque D. (2015). Selective Ligand Activity at Nur/Retinoid X Receptor Complexes Revealed by Dimer-Specific Bioluminescence Resonance Energy Transfer-Based Sensors. FASEB J..

[66] Huang J.K., Jarjour A.A., Nait Oumesmar B., Kerninon C., Williams A., Krezel W., Kagechika H., Bauer J., Zhao C., Baron-Van Evercooren A. (2011). Retinoid X Receptor Gamma Signaling Accelerates CNS Remyelination. Nat. Neurosci..

[67] Clement V., Sanchez P., De Tribolet N., Radovanovic I., I Altaba A.R. (2017). HEDGEHOG-GLI1 signaling regulates human glioma growth, cancer stem cell self-renewal, and tumorigenicity. Curr. Biol..

[68] Liu C., Sage J.C., Miller M.R., Verhaak R.G.W., Hippenmeyer S., Vogel H., Foreman O., Bronson R.T., Nishiyama A., Luo L. (2011). Mosaic analysis with double markers reveals tumor cell of origin in glioma. Cell.

[69] Mastrangelo E., Milani M. (2018). Role and inhibition of GLI1 protein in cancer. Lung Cancer Targets Ther..

[70] Frank-Kamenetsky M., Zhang X.M., Bottega S., Guicherit O., Wichterle H., Dudek H., Bumcrot D., Wang F.Y., Jones S., Shulok J. (2002). Small-Molecule Modulators of Hedgehog Signaling: Identification and Characterization of Smoothened Agonists and Antagonists. J. Biol..

[71] Wang C., Wu H., Katritch V., Han G.W., Huang X.-P., Liu W., Siu F.Y., Roth B.L., Cherezov V., Stevens R.C. (2013). Structure of the Human Smoothened Receptor 7TM Bound to an Antitumor Agent. Nature.

[72] Robarge K.D., Brunton S.A., Castanedo G.M., Cui Y., Dina M.S., Goldsmith R., Gould S.E., Guichert O., Gunzner J.L., Halladay J. (2009). GDC-0449-a Potent Inhibitor of the Hedgehog Pathway. Bioorg. Med. Chem. Lett..

[73] Nachtergaele S., Whalen D.M., Mydock L.K., Zhao Z., Malinauskas T., Krishnan K., Ingham P.W., Covey D.F., Siebold C., Rohatgi R. (2013). Structure and Function of the Smoothened Extracellular Domain in Vertebrate Hedgehog Signaling. eLife.

[74] Huang P., Nedelcu D., Watanabe M., Jao C., Kim Y., Liu J., Salic A. (2016). Cellular Cholesterol Directly Activates Smoothened in Hedgehog Signaling. Cell.

[75] Chen J.K., Taipale J., Cooper M.K., Beachy P.A. (2002). Inhibition of Hedgehog Signaling by Direct Binding of Cyclopamine to Smoothened. Genes Dev..

[76] Kim J., Aftab B.T., Tang J.Y., Kim D., Lee A.H., Rezaee M., Kim J., Chen B., King E.M., Borodovsky A. (2013). Itraconazole and Arsenic Trioxide Inhibit Hedgehog Pathway Activation and Tumor Growth Associated with Acquired Resistance to Smoothened Antagonists. Cancer Cell.

[77] Chen J.K., Taipale J., Young K.E., Maiti T., Beachy P.A. (2002). Small Molecule Modulation of Smoothened Activity. Proc. Natl. Acad. Sci. USA.

[78] Gonnissen A., Isebaert S., Haustermans K. (2015). Targeting the Hedgehog signaling pathway in cancer: Beyond Smoothened. Oncotarget.

[79] Bai C.B., Joyner A.L. (2001). Gli1 Can Rescue the In Vivo Function of Gli2. Development.

[80] Dennler S., André J., Alexaki I., Li A., Magnaldo T., Ten Dijke P., Wang X.-J., Verrecchia F., Mauviel A. (2007). Induction of Sonic Hedgehog Mediators by Transforming Growth Factor-Beta: Smad3-Dependent Activation of Gli2 and Gli1 Expression In Vitro and In Vivo. Cancer Res..

[81] Falcón-Urrutia P., Carrasco C.M., Lois P., Palma V., Roth A.D. (2015). Shh Signaling through the Primary Cilium Modulates Rat Oligodendrocyte Differentiation. PLoS ONE.

[82] Cherry A.L., Finta C., Karlström M., Jin Q., Schwend T., Astorga-Wells J., Zubarev R.A., Del Campo M., Criswell A.R., De Sanctis D. (2013). Structural basis of SUFU-GLI interaction in human Hedgehog signaling regulation. Acta Crystallogr. D Biol. Crystallogr..

[83] Pogoda H.M., Sternheim N., Lyons D.A., Diamond B., Hawkins T.A., Woods I.G., Bhatt D.H., Franzini-Armstrong C., Dominguez C., Arana N. (2006). A Genetic Screen Identifies Genes Essential for Development of Myelinated Axons in Zebrafish. Dev. Biol..

[84] Bai C.B., Stephen D., Joyner A.L. (2004). All Mouse Ventral Spinal Cord Patterning by Hedgehog Is Gli Dependent and Involves an Activator Function of Gli3. Dev. Cell.

[85] Park H.L., Bai C., Platt K.A., Matise M.P., Beeghly A., Hui C.C., Nakashima M., Joyner A.L. (2000). Mouse GLI1 mutants are viable but have defects in SHH signaling in combination with a GLI2 mutation. Development.

[86] Kitaura Y., Hojo H., Komiyama Y., Takato T., Chung U.I., Ohba S., Marie P.J. (2014). GLI1 haploinsufficiency leads to decreased bone mass with an uncoupling of bone metabolismin adult mice. PLoS ONE.

[87] Palencia-Campos A., Ullah A., Nevado J., Yildirim R., Unal E., Ciorraga M., Barruz P., Chico L., Piceci-Sparascio F., Guida V. (2017). GLI1 Inactivation Is Associated with Developmental Phenotypes Overlapping with Ellis-van Creveld Syndrome. Hum. Mol. Genet..

[88] Pan Y., Bai C.B., Joyner A.L., Wang B. (2006). Sonic Hedgehog Signaling Regulates Gli2 Transcriptional Activity by Suppressing Its Processing and Degradation. Mol. Cell. Biol..

[89] Pan Y., Wang C., Wang B. (2009). Phosphorylation of Gli2 by protein kinase A is required for Gli2 processing and degradation and the Sonic Hedgehog-regulated mouse development. Dev Biol..

[90] Vortkamp A., Gessler M., Grzeschik K.H. (1991). GLI3 Zinc-Finger Gene Interrupted by Translocations in Greig Syndrome Families. Nature.

[91] Wild A., Kalff-Suske M., Vortkamp A., Bornholdt D., König R., Grzeschik K.H. (1997). Point Mutations in Human GLI3 Cause Greig Syndrome. Hum. Mol. Genet..

[92] Kong J.H., Yang L., Dessaud E., Chuang K., Moore D.M., Rohatgi R., Briscoe J., Novitch B.G. (2015). Notch Activity Modulates the Responsiveness of Neural Progenitors to Sonic Hedgehog Signaling. Dev. Cell.

[93] Radhakrishna U., Wild A., Grzeschik K.H., Antonarakis S.E. (1997). Mutation in GLI3 in Postaxial Polydactyly Type A. Nat. Genet..

[94] Radhakrishna U., Bornholdt D., Scott H.S., Patel U.C., Rossier C., Engel H., Bottani A., Chandal D., Blouin J.L., Solanki J.V. (1999). The Phenotypic Spectrum of GLI3 Morphopathies Includes Autosomal Dominant Preaxial Polydactyly Type-IV and Postaxial Polydactyly Type-A/B; No Phenotype Prediction from the Position of GLI3 Mutations. Am. J. Hum. Genet..

[95] França M.M., Jorge A.A.L., Carvalho L.R.S., Costalonga E.F., Vasques G.A., Leite C.C., Mendonca B.B., Arnhold I.J.P. (2010). Novel Heterozygous Nonsense GLI2 Mutations in Patients with Hypopituitarism and Ectopic Posterior Pituitary Lobe without Holoprosencephaly. J. Clin. Endocrinol. Metab..

[96] Roessler E., Du Y.-Z., Mullor J.L., Casas E., Allen W.P., Gillessen-Kaesbach G., Roeder E.R., Ming J.E., I Altaba A.R., Muenke M. (2003). Loss-of-Function Mutations in the Human GLI2 Gene Are Associated with Pituitary Anomalies and Holoprosencephaly-like Features. Proc. Natl. Acad. Sci. USA.

[97] Katoh Y., Katoh M. (2009). Integrative Genomic Analyses on GLI1: Positive Regulation of GLI1 by Hedgehog-GLI, TGFbeta-Smads, and RTK-PI3K-AKT Signals, and Negative Regulation of GLI1 by Notch-CSL-HES/HEY, and GPCR-Gs-PKA Signals. Int. J. Oncol..

[98] Stecca B., Mas C., Clement V., Zbinden M., Correa R., Piguet V., Beermann F., I Altaba A.R. (2007). Melanomas Require HEDGEHOG-GLI Signaling Regulated by Interactions between GLI1 and the RAS-MEK/AKT Pathways. Proc. Natl. Acad. Sci. USA.

[99] Seto M., Ohta M., Asaoka Y., Ikenoue T., Tada M., Miyabayashi K., Mohri D., Tanaka Y., Ijichi H., Tateishi K. (2009). Regulation of the Hedgehog Signaling by the Mitogen-Activated Protein Kinase Cascade in Gastric Cancer. Mol. Carcinog..

[100] Nolan-Stevaux O., Lau J., Truitt M.L., Chu G.C., Hebrok M., Fernández-Zapico M.E., Hanahan D. (2009). GLI1 Is Regulated through Smoothened-Independent Mechanisms in Neoplastic Pancreatic Ducts and Mediates PDAC Cell Survival and Transformation. Genes Dev..

[101] Ng J.M.Y., Curran T. (2011). The Hedgehog’s Tale: Developing Strategies for Targeting Cancer. Nat. Rev. Cancer.

[102] Wang Y., Ding Q., Yen C.-J., Xia W., Izzo J.G., Lang J.-Y., Li C.-W., Hsu J.L., Miller S.A., Wang X. (2012). The Crosstalk of MTOR/S6K1 and Hedgehog Pathways. Cancer Cell.

[103] Ji Z., Mei F.C., Xie J., Cheng X. (2007). Oncogenic KRAS activates hedgehog signaling pathway in pancreatic cancer cells. J. Biol. Chem..

[104] Kasper M., Regl G., Eichberger T., Frischauf A.-M., Aberger F. (2007). Efficient Manipulation of Hedgehog/GLI Signaling Using Retroviral Expression Systems. Methods Mol. Biol..

[105] Bercury K.K., Dai J., Sachs H.H., Ahrendsen J.T., Wood T.L., Macklin W.B. (2014). Conditional Ablation of Raptor or Rictor Has Differential Impact on Oligodendrocyte Differentiation and CNS Myelination. J. Neurosci..

[106] Lebrun-Julien F., Bachmann L., Norrmén C., Trötzmüller M., Köfeler H., Rüegg M.A., Hall M.N., Suter U. (2014). Balanced MTORC1 Activity in Oligodendrocytes Is Required for Accurate CNS Myelination. J. Neurosci..

[107] Wahl S.E., McLane L.E., Bercury K.K., Macklin W.B., Wood T.L. (2014). Mammalian Target of Rapamycin Promotes Oligodendrocyte Differentiation, Initiation and Extent of CNS Myelination. J. Neurosci..

[108] Zou Y., Jiang W., Wang J., Li Z., Zhang J., Bu J., Zou J., Zhou L., Yu S., Cui Y. (2014). Oligodendrocyte Precursor Cell-Intrinsic Effect of Rheb1 Controls Differentiation and Mediates MTORC1-Dependent Myelination in Brain. J. Neurosci..

[109] Tyler W.A., Gangoli N., Gokina P., Kim H.A., Covey M., Levison S.W., Wood T.L. (2009). Activation of the Mammalian Target of Rapamycin (MTOR) Is Essential for Oligodendrocyte Differentiation. J. Neurosci..

[110] Aronova S., Wedaman K., Aronov P.A., Fontes K., Ramos K., Hammock B.D., Powers T. (2008). Regulation of Ceramide Biosynthesis by TOR Complex 2. Cell Metab..

[111] Ingham P.W., Nakano Y., Seger C. (2011). Mechanisms and Functions of Hedgehog Signaling across the Metazoa. Nat. Rev. Genet..

[112] Riobó N.A., Lu K., Ai X., Haines G.M., Emerson C.P. (2006). Phosphoinositide 3-Kinase and Akt Are Essential for Sonic Hedgehog Signaling. Proc. Natl. Acad. Sci. USA.

[113] Li Y.-H., Luo J., Mosley Y.-Y.C., Hedrick V.E., Paul L.N., Chang J., Zhang G., Wang Y.-K., Banko M.R., Brunet A. (2015). AMP-Activated Protein Kinase Directly Phosphorylates and Destabilizes Hedgehog Pathway Transcription Factor GLI1 in Medulloblastoma. Cell Rep..

[114] Ma X., Drannik A., Jiang F., Peterson R., Turnbull J. (2017). Crosstalk between Notch and Sonic Hedgehog Signaling in a Mouse Model of Amyotrophic Lateral Sclerosis. Neuroreport.

[115] Juryńczyk M., Jurewicz A., Bielecki B., Raine C.S., Selmaj K. (2005). Inhibition of Notch Signaling Enhances Tissue Repair in an Animal Model of Multiple Sclerosis. J. Neuroimmunol..

[116] Juryńczyk M., Selmaj K. (2010). Notch: A New Player in MS Mechanisms. J. Neuroimmunol..

[117] Saher G., Brügger B., Lappe-Siefke C., Möbius W., Tozawa R., Wehr M.C., Wieland F., Ishibashi S., Nave K.-A. (2005). High Cholesterol Level Is Essential for Myelin Membrane Growth. Nat. Neurosci..

[118] Vallett S.M., Sanchez H.B., Rosenfeld J.M., Osborne T.F. (1996). A Direct Role for Sterol Regulatory Element Binding Protein in Activation of 3-Hydroxy-3-Methylglutaryl Coenzyme A Reductase Gene. J. Biol. Chem..

[119] Bennett M.K., Toth J.I., Osborne T.F. (2004). Selective association of sterol regulatory element-binding protein isoforms with target promoters in vivo. J. Biol. Chem..

[120] Tabor D.E., Kim J.B., Spiegelman B.M., Edwards P.A. (1999). Identification of Conserved Cis-Elements and Transcription Factors Required for Sterol-Regulated Transcription of Stearoyl-CoA Desaturase 1 and 2. J. Biol. Chem..

[121] Halder S.K., Fink M., Waterman M.R., Rozman D. (2002). A CAMP-Responsive Element Binding Site Is Essential for Sterol Regulation of the Human Lanosterol 14alpha-Demethylase Gene (CYP51). Mol. Endocrinol..

[122] Ikeda Y., Yamamoto J., Okamura M., Fujino T., Takahashi S., Takeuchi K., Osborne T.T., Yamamoto T.T. (2001). Transcriptional regulation of the murine acetyl CoA synthetase 1 gene through multiple clustered binding sites for SREBPs and a single neighboring site for Sp1. J Biol. Chem..

[123] Prabhu A.V., Sharpe L.J., Brown A.J. (2014). The Sterol-Based Transcriptional Control of Human 7-Dehydrocholesterol Reductase (DHCR7): Evidence of a Cooperative Regulatory Program in Cholesterol Synthesis. Biochim. Biophys. Acta.

[124] Ye J., DeBose-Boyd R.A. (2011). Regulation of Cholesterol and Fatty Acid Synthesis. Cold Spring Harb. Perspect. Biol..

[125] Norrmèn C., Figlia G., Lebrun-Julien F., Pereira J.A., Trötzmüller M., Köfeler H.C., Rantanen V., Wessig C., Van Deijk A.-L.F., Smit A.B. (2014). MTORC1 Controls PNS Myelination along the MTORC1-RxRγ-SREBP-Lipid Biosynthesis Axis in Schwann Cells. Cell Rep..

[126] Saher G., Quintes S., Möbius W., Wehr M.C., Krämer-Albers E.-M., Brügger B., Nave K.-A. (2009). Cholesterol Regulates the Endoplasmic Reticulum Exit of the Major Membrane Protein P0 Required for Peripheral Myelin Compaction. J. Neurosci..

[127] Porstmann T., Santos C.R., Griffiths B., Cully M., Wu M., Leevers S., Griffiths J.R., Chung Y.-L., Schulze A. (2008). SREBP Activity Is Regulated by MTORC1 and Contributes to Akt-Dependent Cell Growth. Cell Metab..

[128] Düvel K., Yecies J.L., Menon S., Raman P., Lipovsky A.I., Souza A.L., Triantafellow E., Ma Q., Gorski R., Cleaver S. (2010). Activation of a Metabolic Gene Regulatory Network Downstream of MTOR Complex 1. Mol. Cell.

[129] Guerrero I., Kornberg T.B. (2014). Hedgehog and Its Circuitous Journey from Producing to Target Cells. Semin. Cell Dev. Biol..

[130] Qi X., Schmiege P., Coutavas E., Wang J., Li X. (2018). Structures of Human Patched and Its Complex with Native Palmitoylated Sonic Hedgehog. Nature.

[131] Carstea E.D., Morris J.A., Coleman K.G., Loftus S.K., Zhang D., Cummings C., Gu J., Rosenfeld M.A., Pavan W.J., Krizman D.B. (1997). Niemann-Pick C1 Disease Gene: Homology to Mediators of Cholesterol Homeostasis. Science.

[132] Bidet M., Joubert O., Lacombe B., Ciantar M., Nehmé R., Mollat P., Brétillon L., Faure H., Bittman R., Ruat M. (2011). The Hedgehog Receptor Patched Is Involved in Cholesterol Transport. PLoS ONE.

[133] Yu T., Lieberman A.P. (2013). Npc1 Acting in Neurons and Glia Is Essential for the Formation and Maintenance of CNS Myelin. PLoS Genet..

[134] Incardona J.P., Gruenberg J., Roelink H. (2002). Sonic Hedgehog Induces the Segregation of Patched and Smoothened in Endosomes. Curr. Biol..

[135] Khaliullina H., Panáková D., Eugster C., Riedel F., Carvalho M., Eaton S. (2009). Patched Regulates Smoothened Trafficking Using Lipoprotein-Derived Lipids. Development.

[136] Blassberg R., Macrae J.I., Briscoe J., Jacob J. (2016). Reduced Cholesterol Levels Impair Smoothened Activation in Smith-Lemli-Opitz Syndrome. Hum. Mol. Genet..

[137] Byrne E.F.X., Sircar R., Miller P.S., Hedger G., Luchetti G., Nachtergaele S., Tully M.D., Mydock-McGrane L., Covey D.F., Rambo R.P. (2016). Structural Basis of Smoothened Regulation by Its Extracellular Domains. Nature.

[138] Furtado L.V., Kelley R.I., Opitz J.M. (2016). Disorders of Sterol Biosynthesis. Transl. Sci. Rare Dis..

